# Comparative Retrospective Evaluation of the Clinical and Mycological Efficacy of 69% Nitric Acid, 1064 nm Nd:YAG Laser, and Their Combination in the Treatment of *Trichophyton rubrum* Onychomycosis over a 12-Month Follow-Up

**DOI:** 10.3390/idr18010001

**Published:** 2025-12-20

**Authors:** Raquel García De La Peña, José María Juárez-Jiménez, João Miguel Costa Martiniano, Ana María Rayo Pérez

**Affiliations:** 1 Department of Podiatry, University of Seville, 41009 Seville, Spain; raquelgp@us.es (R.G.D.L.P.); jmjuarez@us.es (J.M.J.-J.); 2 Portuguese Red Cross Higher School of Health, University of Lisbon, 1350-125 Lisbon, Portugal; jmartiniano@esscvp.eu

**Keywords:** onychomycosis, *Trichophyton rubrum*, Nd:YAG laser, nitric acid, combination therapy

## Abstract

**Background**: Onychomycosis is a common nail infection primarily caused by *Trichophyton rubrum*, posing therapeutic challenges due to poor antifungal penetration and high recurrence rates. Conventional treatments include topical and systemic antifungals, but novel approaches such as laser therapy and chemical agents like nitric acid have emerged as promising alternatives or adjuncts. However, comparative evidence regarding the clinical and mycological efficacy of these treatments remains limited. **Objectives**: We aimed to assess and compare the clinical and mycological efficacy of three therapeutic modalities—69% nitric acid, 1064 nm Nd:YAG laser, and their combination—in the treatment of *Trichophyton rubrum* onychomycosis over a 12-month follow-up period. **Methods**: A prospective, comparative, observational study was conducted, assigning patients with confirmed onychomycosis to one of three treatment groups: nitric acid, Nd:YAG 1064 nm laser, or combination therapy. Clinical and mycological cure rates, mean time to clinical resolution, changes in Onychomycosis Severity Index [OSI] scores, and mycological relapse rates were assessed over a 12-month follow-up. **Results**: All three groups demonstrated significant improvement in both clinical and mycological cure rates, with the combination therapy yielding the most favorable outcomes in terms of response speed and durability. Laser and nitric acid monotherapies were also effective, though associated with lower cure rates and longer times to resolution. The relapse rate was lowest in the combination group. **Conclusions**: The combination of nitric acid and Nd:YAG laser appears to be a more effective therapeutic option for *Trichophyton rubrum* onychomycosis, offering superior clinical and mycological outcomes compared to monotherapies, with faster resolution and lower relapse rates. These findings suggest that combination therapy may optimize the management of this challenging nail infection.

## 1. Introduction

Onychomycosis is a chronic fungal infection affecting the nail unit, predominantly the toenails, and accounts for approximately 50% of all nail disorders worldwide [1]. It is most commonly caused by dermatophytes—particularly *Trichophyton rubrum*—although yeasts and non-dermatophyte molds may also be involved. Clinically, onychomycosis is characterized by nail discoloration, thickening, fragility, and dystrophy, often leading to functional impairment and a significant reduction in quality of life [2].

The prevalence of onychomycosis has increased over recent decades, driven by factors such as aging populations, diabetes mellitus, immunosuppression, and prolonged use of occlusive footwear [3]. Accurate diagnosis is essential to guide appropriate therapy and avoid unnecessary or ineffective treatments. However, diagnosis remains challenging, as clinical features may mimic other nail disorders and conventional diagnostic techniques—such as potassium hydroxide (KOH) microscopy and fungal culture—exhibit variable sensitivity [4,5].

Therapeutic management of onychomycosis is complex due to the slow growth of the nail plate and the difficulty of achieving sustained antifungal concentrations within the nail unit. Systemic antifungal agents, including terbinafine and itraconazole, remain first-line therapies due to their efficacy; however, their use may be limited by potential adverse effects, drug interactions, and contraindications [1,6]. Topical therapies offer a superior safety profile but are often less effective in moderate-to-severe disease because of poor nail plate penetration [7,8].

In recent years, novel topical antifungals with improved penetration characteristics—such as efinaconazole, tavaborole, and luliconazole—have expanded available treatment options [7,9]. In parallel, physical modalities, particularly laser-based therapies, have gained interest as alternative or adjunctive treatments. Among these, the 1064 nm Nd:YAG laser has been proposed to exert antifungal effects through selective photothermal mechanisms that damage fungal structures while sparing the surrounding tissue [10,11,12,13]. Nevertheless, heterogeneity in laser parameters, treatment protocols, and outcome measures has resulted in inconsistent efficacy reports and limited consensus regarding its clinical role [5,14].

Combination strategies integrating physical and chemical therapies have, therefore, emerged as a potential means to overcome the limitations of monotherapies. Nitric acid exhibits antimicrobial, keratolytic, and protein-denaturing properties that may facilitate nail plate permeability and enhance fungal eradication. When combined with Nd:YAG laser therapy, a synergistic effect may occur through increased penetration, localized thermal damage to fungal elements, and disruption of fungal biofilms and spores [8,9,15]. From a physiological perspective, laser-induced heating may alter fungal cell membranes and mitochondrial function, while nitric acid contributes to local chemical denaturation, potentially amplifying antifungal efficacy.

Despite therapeutic advances, recurrence remains a major challenge in onychomycosis management. Long-term success depends not only on initial pathogen eradication but also on sustained suppression of residual fungal elements and appropriate follow-up strategies [1,6].

The primary objective of this study was to evaluate and compare the clinical and mycological efficacy of three therapeutic modalities—69% nitric acid, 1064 nm Nd:YAG laser, and their combination—in the treatment of *Trichophyton rubrum* onychomycosis over a 12-month follow-up period. Secondary objectives included assessment of time to clinical resolution, mycological relapse rates, and changes in the Onychomycosis Severity Index (OSI), as well as exploration of the relationship between baseline disease severity and therapeutic response.

## 2. Materials and Methods

### 2.1. Study Design

An observational, retrospective, comparative study was conducted with a 12-month follow-up. Data were obtained through a review of medical records and standardized photographic documentation of patients treated between January 2020 and December 2023 at a specialized dermatology clinic.

### 2.2. Population and Sample

A total of 90 patients with confirmed *Trichophyton rubrum* onychomycosis were included using non-probability convenience sampling. Patients were categorized according to the treatment they had received as part of routine clinical practice:

- Group A: 69% nitric acid (monotherapy (*n* = 30);
- Group B: 1064 nm Nd:YAG laser monotherapy (*n* = 30);
- Group C: combined 69% nitric acid + 1064 nm Nd:YAG laser therapy (*n* = 30).

As this was a retrospective study, treatment allocation was not controlled by the investigators and reflected clinical decision-making at the time of care.

### 2.3. Eligibility Criteria

Inclusion criteria were confirmed diagnosis by positive KOH microscopy and fungal culture for *T. rubrum*; exclusive involvement of a single hallux nail; completion of one of the three treatment modalities; availability of complete clinical, mycological, and photographic documentation; and a minimum follow-up of 12 months.

Exclusion criteria included immunosuppression, uncontrolled systemic disease, antifungal treatment within the preceding six months, bilateral nail involvement, mixed fungal infections, and incomplete records.

### 2.4. Severity Assessment

Disease severity was assessed using the Onychomycosis Severity Index (OSI) and classified as follows:

- Mild: OSI 1–5;
- Moderate: OSI 6–15;
- Severe: OSI > 15.

Two independent dermatologists evaluated baseline severity using standardized clinical and photographic records.

### 2.5. Therapeutic Interventions

**Figure 1:**
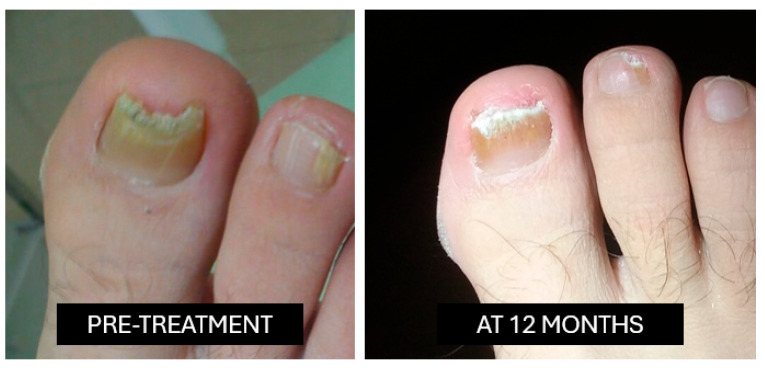
Nitric acid.

**Nitric acid (69%)**: This is applied once weekly for four weeks following standardized superficial nail abrasion performed with a sterile abrasive instrument, limited to the dorsal nail plate (Figure 1).

**Figure 2:**
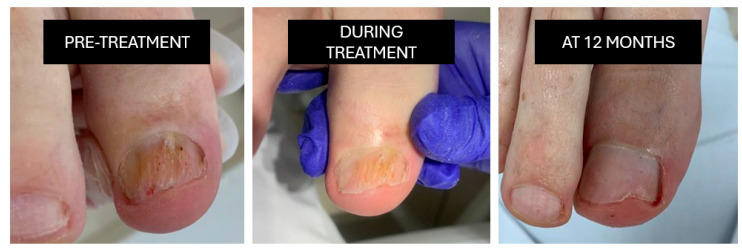
Nd:YAG laser.

**Nd:YAG laser (1064 nm; S30 Podylas™, INTERmedic, Cerdanyola del Vallès, Barcelona, Spain)**: Three sessions are conducted at two-week intervals (35–40 J/cm^2^, long-pulse mode), applied uniformly across the nail plate Figure 2).

**Combined therapy**: Nitric acid is applied during the first week, followed by the first laser session within the same week; two additional laser sessions follow at two-week intervals.

### 2.6. Safety Monitoring

Clinical records were reviewed for adverse events, including nail plate damage, periungual ulceration, pain, infection, or pigmentary changes. Events were classified as mild, moderate, or severe.

### 2.7. Study Variables

The study variables included one independent variable—the type of treatment received—and several dependent variables related to clinical and mycological outcomes. The dependent variables comprised clinical cure, defined as the visual restoration of normal nail appearance; mycological cure, determined by negative culture and KOH results; and the average time to clinical resolution, measured in weeks. Additionally, mycological relapse was assessed based on the recurrence of clinical signs accompanied by positive mycological findings at the 12-month follow-up. The baseline Onychomycosis Severity Index [OSI] score was also recorded to correlate initial disease severity with treatment response.

### 2.8. Data Collection

A retrospective review was conducted of clinical records, mycological results, and photographic documentation. Data were entered into an anonymized database. Clinical and mycological assessments were recorded at treatment initiation, at 12 weeks [immediate post-treatment], and at 12 months [long-term follow-up].

### 2.9. Statistical Analysis

Statistical analysis was performed using SPSS version 25.0 IBM Corp., Armonk, NY, USA). Descriptive statistics were used for both quantitative and qualitative variables. Group comparisons were conducted as follows:

- ANOVA or Kruskal–Wallis test for comparing means [OSI score, time to resolution].
- Chi-square test or Fisher’s exact test for categorical variables [clinical cure, mycological cure, and relapse rates].
- A *p*-value < 0.05 was considered statistically significant.

### 2.10. Ethical Considerations

Due to the retrospective nature of the study, individual informed consent was not required. Data confidentiality and anonymity were strictly maintained in accordance with national regulations and the principles outlined in the Declaration of Helsinki.

## 3. Results

### 3.1. Descriptive Statistics

#### 3.1.1. Demographic Variables

**Age Distribution**

The total sample (*n* = 90) had a mean age of 47.3 ± 8.9 years (range: 33–63). Group means were LASER 47.1 ± 8.7, NITRIC 47.6 ± 9.2, and COMBINED 47.2 ± 8.9. The Shapiro–Wilk test indicated normal distribution (W = 0.986, *p* = 0.432), allowing parametric comparisons.

**Sex Distribution**

Women represented 66.7% of the sample and men 33.3%. Distribution was similar across groups: LASER 63.3%, NITRIC 67.9%, COMBINED 69.2% (χ^2^ = 0.832, *p* = 0.832), confirming baseline homogeneity.

#### 3.1.2. Baseline Clinical Variables

**Location of Nail Involvement**

Involvement was 56% right hallux and 44% left hallux, with no significant lateral predominance (*p* > 0.05).

**Baseline Severity (OSI Classification)**

Patients were classified as mild (4.8%, OSI < 8), moderate (83.3%, OSI 8–15), and severe (11.9%, OSI > 15). Distribution among groups was homogeneous (*p* = 0.943). Mean baseline OSI: LASER 11.2 ± 3.1, NITRIC 11.3 ± 3.8, COMBINED 11.5 ± 3.6. OSI scores were non-normally distributed (Shapiro–Wilk W = 0.962, *p* = 0.012), so non-parametric tests were applied.

### 3.2. Post-Treatment Outcomes

#### Clinical Efficacy

**OSI Reduction**

All groups (Table 1) showed significant OSI reduction (Kruskal–Wallis χ^2^ = 18.74, *p* < 0.001):

Post hoc analysis: COMBINED > LASER (*p* < 0.001, d = 1.45) and COMBINED > NITRIC (*p* = 0.003, d = 0.89). NITRIC > LASER (*p* = 0.021, d = 0.56).

**Time to Resolution** (Table 2)

Kruskal–Wallis χ^2^ = 12.56, *p* = 0.002. Post hoc: COMBINED < LASER (*p* = 0.001, d = 1.12), COMBINED < NITRIC (*p* = 0.047, d = 0.52).

**Cure Rates** (Table 3)

Odds ratio COMBINED vs. LASER for mycological cure: 4.23 (95% CI: 1.45–12.34). Relative risk reduction in relapse COMBINED vs. LASER 64.4%.

### 3.3. Correlation Analysis

- Baseline OSI correlated with time to resolution (ρ = 0.62, *p* < 0.001); each OSI point added 1.2 weeks (R^2^ = 0.38).
- Post-treatment OSI correlated with relapse (ρ = 0.51, *p* < 0.001); OSI >5 increased relapse risk 3.8-fold (95% CI: 2.1–6.9).
- No significant correlations: age vs. cure (ρ = –0.18, *p* = 0.087), sex vs. treatment response (*p* = 0.214).

### 3.4. Effect Size

Effect size (Table 4) measures were calculated to quantify the magnitude of the observed effects and to complement statistical significance testing. Eta squared (η^2^) and Cohen’s *d* were used for continuous variables, while Cramer’s V was applied to categorical outcomes. Effect sizes were interpreted according to conventional thresholds to facilitate the clinical interpretation of the results beyond *p*-values.

### 3.5. Subgroup Analysis by Baseline Severity

A subgroup analysis (Table 5) was performed to evaluate treatment effectiveness according to baseline severity. Outcomes were compared across severity categories to explore potential differences in response and to assess the consistency of the treatment effect among subgroups.

### 3.6. Safety and Limitations

No serious adverse events were reported in any group. Mild, transient erythema or burning sensation was observed in <10% of patients in the nitric acid and COMBINED groups, resolving spontaneously.

Limitations include the retrospective design, single-center setting, convenience sampling, and small sample size for mild cases, which may limit generalizability. The study only included hallux nails, and only one nail per patient was analyzed. Future prospective, randomized studies are recommended to confirm these findings and standardize protocols.

## 4. Discussion

This study evaluated the clinical and mycological efficacy of three therapeutic modalities—69% nitric acid, 1064 nm Nd:YAG laser, and their combination—in the treatment of *Trichophyton rubrum* onychomycosis over a 12-month follow-up. The results suggest that combined therapy may provide superior outcomes compared with monotherapies, including greater reductions in the Onychomycosis Severity Index (OSI), higher clinical and mycological cure rates, shorter time to clinical resolution, and lower relapse rates. These findings support the potential value of multimodal strategies in managing persistent onychomycosis.

The observed benefits of combined therapy are likely due to complementary mechanisms. Nitric acid, with its antimicrobial and keratolytic properties, may enhance nail plate permeability and facilitate fungal eradication, while the 1064 nm Nd:YAG laser induces localized thermal effects that selectively damage fungal structures without harming surrounding tissue. The synergistic effect of chemical and photothermal actions may explain the faster OSI reduction and higher proportion of patients achieving clinical and mycological cure. These findings are consistent with previous studies indicating that combination approaches can enhance antifungal penetration and efficacy compared with single modalities [16,17,18,19,20].

The combined therapy group also demonstrated a clinically relevant reduction in time to resolution, with a mean of 14.8 weeks compared to 15.9 and 17.6 weeks in the nitric acid and laser groups, respectively. Shorter treatment durations may improve patient adherence and satisfaction and could potentially reduce the cumulative costs and risks associated with prolonged therapies; this is particularly relevant in chronic infections such as onychomycosis [21].

Relapse rates were lower in the combined therapy group (15.4%) compared to monotherapies. This may reflect more complete fungal eradication through the complementary mechanisms of chemical debridement and photothermal destruction. Additionally, the study confirmed that higher post-treatment OSI scores are associated with an increased risk of relapse, emphasizing the importance of achieving a thorough therapeutic response to minimize recurrence [22].

Baseline homogeneity in demographic and clinical variables—including age, sex, nail location, and initial severity—strengthens the comparative validity of the findings. However, the retrospective, observational design and sample size—particularly in the mild OSI subgroup—limit the generalizability of results. Randomized, controlled studies with larger samples are required to confirm these findings and better assess efficacy across all severity levels [5,23]. Furthermore, the 12-month follow-up, while longer than in many previous studies, may not capture all late recurrences, underscoring the need for extended monitoring in future research.

In terms of safety, no serious adverse events were reported in any group, aligning with literature describing good tolerability for both Nd:YAG laser and nitric acid [21,24,25]. Mild, transient local effects were observed but did not necessitate treatment discontinuation. These observations suggest that combined therapy can be safely applied under appropriate clinical supervision, though systematic safety monitoring is recommended [26,27,28,29].

From a therapeutic perspective, these findings support the integration of physical and chemical modalities into personalized treatment protocols, tailored to disease severity and patient characteristics. Monotherapies, particularly laser treatment, may be more appropriate for selected cases or as adjuncts to combination strategies to optimize outcomes [5,25]. Moreover, the significant impact of onychomycosis on quality of life highlights the clinical relevance of effective, sustained treatment, which the combined therapy appears to facilitate [24].

Finally, the correlation between baseline OSI and therapeutic response reinforces the need for accurate severity assessment when planning individualized treatment strategies. Patients with higher OSI scores may benefit from more intensive or combination therapies to achieve optimal and durable outcomes.

## 5. Conclusions

This retrospective observational study evaluated the clinical and mycological efficacy of three therapeutic modalities—69% nitric acid, 1064 nm Nd:YAG laser, and their combination—in the treatment of *Trichophyton rubrum* onychomycosis over a 12-month follow-up period. The combined therapy demonstrated the highest rates of clinical and mycological cure, with shorter time to resolution, lower relapse rates, and greater improvements in the Onychomycosis Severity Index (OSI) compared with monotherapies.

These findings suggest that the synergistic use of nitric acid and the Nd:YAG laser may provide enhanced and more durable therapeutic outcomes, particularly in moderate-to-severe cases. However, given the retrospective design, limited sample size, and single-center setting, prospective randomized studies are needed to confirm the results and establish standardized protocols for combination therapy in clinical practice.

## Figures and Tables

**Table 1:** OSI Reduction.

Group	Baseline OSI	Post-Treatment OSI	Absolute Reduction	% Reduction
LASER	11.2 ± 3.1	4.5 ± 2.2	6.7	59.8%
NITRIC	11.3 ± 3.8	3.1 ± 2.0	8.2	72.6%
COMBINED	11.5 ± 3.6	1.5 ± 1.1	10.0	87.0%

**Table 2:** Time to resolution.

Group	Mean ± SD (Weeks)
LASER	17.6 ± 2.8
NITRIC	15.9 ± 2.4
COMBINED	14.8 ± 2.2

**Table 3:** Cure rates.

Group	Clinical Cure (%)	Mycological Cure (%)	6-Month Relapse (%)
LASER	70	50	43.3
NITRIC	75	64.3	32.1
COMBINED	88.5	80.8	15.4

**Table 4:** Effect size.

Variable	η^2^	Cohen’s *d*	Interpretation
OSI post-treatment	0.32	1.45	Large
Time to resolution	0.25	1.12	Large
Mycological cure	—	—	Cramer’s V = 0.31 (Moderate)

**Table 5:** Baseline severity.

Severity	Group	% Cure	*p*-Value
Mild	COMBINED	100%	0.021
Moderate	COMBINED	86.4%	<0.001
Severe	COMBINED	75.0%	0.032

## Data Availability

The data supporting the findings of this study are available from the corresponding author upon reasonable request. Due to privacy and ethical restrictions, raw patient data are not publicly available.
